# Molecular Distributions of Soluble Oxidation Products from Coals Characterized by Mass Spectrometers

**DOI:** 10.1155/2018/5174172

**Published:** 2018-04-11

**Authors:** Xing Fan, Fei Wang

**Affiliations:** Key Laboratory of Coal Processing and Efficient Utilization, Ministry of Education, University of Mining & Technology, Xuzhou, Jiangsu 221116, China

## Abstract

Oxidation of three coals with rank from lignite to anthracite in NaOCl aqueous solution was investigated in this study. The oxidation products were characterized by using gas chromatography/mass spectrometry and direct analysis in real-time mass spectrometry. The results showed that most of organic compounds in coals were converted into water-soluble species under mild conditions, even the anthracite. Benzene polycarboxylic acids (BPCAs) and chloro-substituted alkanoic acids (CSAAs) were major products from the reactions. The products from lower rank coals consist of considerable CSAAs and most products from high rank coals are BPCAs. As coal rank increases, the yield of BPCAs with more carboxylic groups increases.

## 1. Introduction

Coal consists of three-dimensional crosslinking networks, commonly called coal macromolecules, with a wide range of small organic species scattered inside the networks [1, 2]. As a nonrenewable fossil fuel, most of coals are used for combustion to acquire electric power and heat [3, 4]. However, carbon emission and acid rain mostly induced by coal burning led us to reconsider the utilization of coal in clean and highly efficient ways [5, 6]. For the high contents of aromatics and organic heteroatom-containing compounds, coal can be utilized for nonfuel purposes such as feedstock for value-added chemicals [7]. However, due to the limit of knowledge on the compositions and structures of coal, nonfuel utilization of coal is still with low efficiency.

Coal oxidation is one of the effective methods for understanding coal structure, especially the composition of organic compounds in coal [7, 8]. Organic acids were obtained from coals by oxidation in alkali or acid solution [9, 10]. Mae et al. [11] reported that oxidation of low rank coals with large amounts yields small molecule fatty acids, such as malonic acid, glycolic acid, formic acid, acetic acid, and oxalic acid. Pietrzak and Wachowska [12] oxidized coals with different ranks using CH_3_COOH and found that a significant part of the organic components of coal was converted into acid soluble products. Oshika and Okuwaki [13] examined the O_2_ oxidation of coal-tar pitch in alkaline solution and found that plenty of benzene polycarboxylic acids were produced. However, these attempts were not successful enough because most of the reactions were performed under severe conditions such as high temperature, high pressure, and strong acids, or only available for low rank coals. Mild oxidation was proven as an effective method to study coal structures and acquire chemicals like aliphatic acids (AAs), benzenepolycarboxylic acids (BPCAs), and other oxygen-containing species from coals [14]. NaOCl is attractive as an industrial oxidant because of its easy availability, environmental friendliness, electrolytic renewability, and low cost [15].

Gas chromatography/mass spectrometry (GC/MS) is a powerful analytical method obtaining the molecular-level information of complex mixture such as biomass and coal derivatives and plays a crucial role in understanding composition and structure of coal [16, 17]. Sample needs to be gasified before injecting into the ion source for ionization. Therefore, GC/MS is designed for the analysis of small, volatile, and thermally stable components within complex mixtures [18]. Direct analysis in real time (DART), an ambient ionization technique, is a potential choice to ionize nonvolatile and thermally labile samples in complex mixtures [19, 20]. Unlike GC/MS, DART-MS allows noncontact and direct analysis of samples in solid, liquid, and gel-based state with minimal sample preparation [21]. DART-MS has attracted attention from various research fields and recently has been applied in the rapid characterization of coal degradation products [22].

In this study, NaOCl was used to oxidize three kinds of Chinese coals, a lignite, a subbituminous coal, and an anthracite. Coals at different ranks have various molecular compositions and structures. Soluble oxidation products were analyzed using GC/MS for understanding the structure difference among various ranks and finding a potential way to obtain organic acids from coals. DART-MS was also applied to obtain an overall molecular information for the oxidation products.

## 2. Experimental

### 2.1. Samples and Reagents

The three coals with various ranks, a lignite, a subbituminous coal, and an anthracite, were collected from Shengli, Shenfu, and Jincheng coal mines, China, and are abbreviated as SLL, SFB, and JCA, respectively. Coal samples were pulverized to pass through a 200-mesh screen (particle size < 75 *μ*m), followed by drying at 80°C in a vacuum oven for 24 h.  Table 1 shows the proximate and ultimate analyses of these coal samples. CH_2_N_2_, (CH_3_CH_2_)_2_O, CH_2_Cl_2_, HCl, NaOCl (6% available chlorine), and anhydrous MgSO_4_ were used in the experiments.

### 2.2. Instruments

A Büchi R-134 rotary evaporator was used for distillation of solvents from the reaction mixture. The instrument for qualitative and quantitative analysis of products is a gas chromatography mass spectrometer (Agilent 6890/5973, USA). The GC is equipped with a capillary column coated with HP-5 (cross-link 5% PH ME siloxane, 30 m length, 0.25 mm inner diameter, and 0.25 *μ*m film thickness). The MS is operated in electron impact (70 eV) mode and a quadrupole analyzer is used as the mass analyzer. The mass range was scanned from 30 to 500 Da. Data were acquired and processed using Chemstation software. The compounds were identified by comparing mass spectra with the NIST05 library data. Quantitative analysis was also performed by GC/MS using a series of authentic compounds as external standards, for example, methyl caproate for monocarboxylic acids (MCAs), dimethyl adipate for dicarboxylic acids (DCAs) and tricarboxylic acids, and dimethyl phthalate for benzene carboxylic acids (BCAs).

A time-of-flight MS (TOF-MS, Model G6210; Agilent Technologies, USA) coupled with a DART (SVP 100; IonSense, Inc., USA) ion source was used to analyze the extracts in positive mode. High purity helium (99.999%, 350°C) was used as auxiliary gas with a flow rate of 2 L min^−1^ and nitrogen gas was used in the standby mode. Parameters of DART were set as follows: capillary voltage 4 kV, collision voltage 175 V, and cone voltage 65 V. The mass spectra were scanned in the *m*/*z* range of 100–1000 and data were processed by MassHunter Data Acquisition. All of the TOF-MS data were processed by the Agilent MassHunter WorkStation Software Qualitative Analysis (Version B.01.03).

### 2.3. Oxidation Procedure and Data Processing

**Figure 1:**
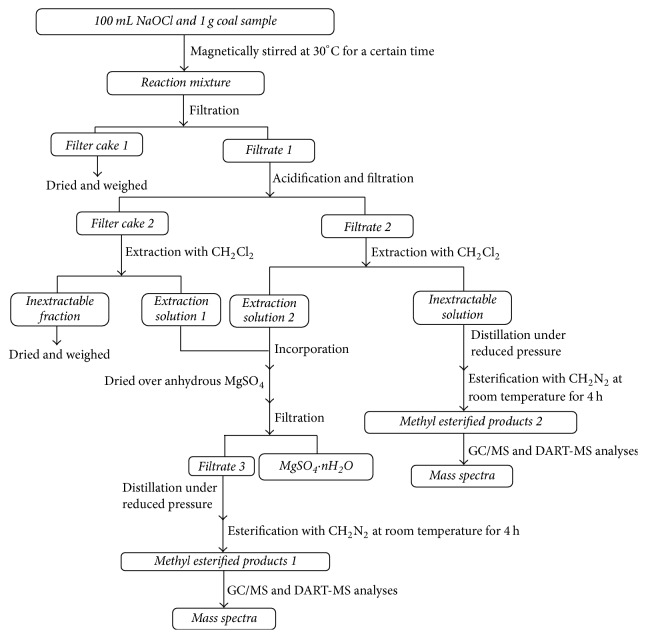
Experimental procedure and analytical methods.

As Figure 1 shows, coal sample (1 g) and NaOCl aqueous solution (100 mL) were added to a 250 mL spherical flask and fully mixed by magnetically stirring at 30°C for a certain time. The reaction mixture was filtrated to obtain filter cake 1 (FC_1_) and filtrate 1 (F_1_). The FC_1_ was dried in a vacuum oven at 80°C for 24 h and then weighed. The F_1_ was acidified with aqueous HCl to pH 2-3 and filtrated to obtain filter cake 2 (FC_2_) and filtrate 2 (F_2_). Both FC_2_ and F_2_ were extracted with CH_2_Cl_2_ to acquire extraction solution 1 (ES_1_) and extraction solution 2 (ES_2_), respectively. ES_1_ and ES_2_ were incorporated to get a mixed solution, which was dried over anhydrous MgSO_4_ and filtrated to get filtrate 3 (F_3_). Then a rotary evaporator under reduced pressure was used to remove CH_2_Cl_2_ from F_3_. The reaction products were esterified with excess CH_2_N_2_ in (CH_3_CH_2_)_2_O solvent to get methyl esterified products 1 (MEPs_1_). The CH_2_Cl_2_-inextractable fraction (IEF) from FC_2_ was also dried and weighed. Water and small amount of CH_2_Cl_2_ in the CH_2_Cl_2_-inextractable solution (IES) from F_2_ were removed with the rotary evaporator under reduced pressure followed by esterification with CH_2_N_2_ in (CH_3_CH_2_)_2_O to afford corresponding methyl esterified products 2 (MEPs_2_). Both MEPs_1_ and MEPs_2_ were analyzed using GC/MS and DART-MS.

## 3. Results and Discussion

The color of reaction mixture for SLL and SFB changed from dark brown to yellow after oxidation with NaOCl for 24 h, but a much longer reaction time (168 h) was needed for JCA. One of the major reasons for such a long reaction time is the difficulty for NaOCl aqueous solution penetration into highly condensed aromatic network of JCA, which induces much lower reactivity for JCA compared with SLL and SFB.  Table 2 lists the yields of FC_1_ and IEF. The low yields of these residues indicate that most of organic compounds in coals are converted to water-soluble species, even the JCA.

**Figure 2:**
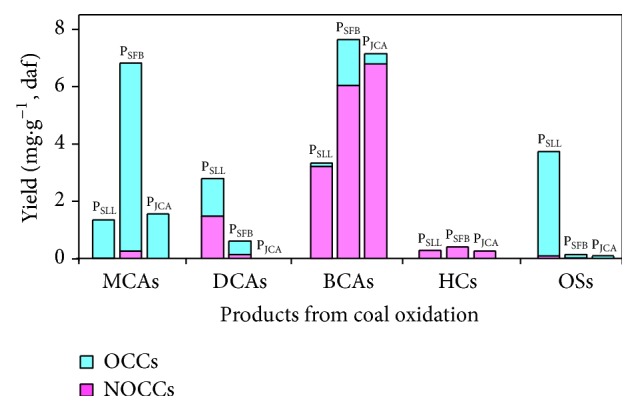
Distribution of products from coal oxidation.

**Figure 3:**
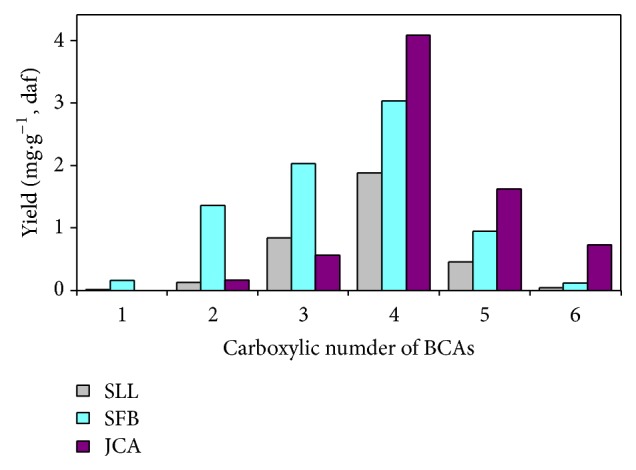
Distribution of BCAs from coal oxidation.

**Figure 4:**
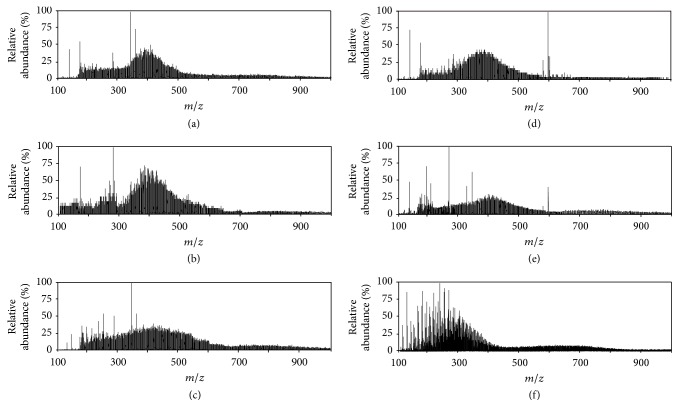
Mass spectra of (a) MEPs_1_-SLL, (b) MEPs_1_-SFB, (c) MEPs_1_-JCA, (d) MEPs_2_-SLL, (e) MEPs_2_-SFB, and (f) MEPs_2_-JCA, obtained by DART-TOF-MS.

As shown in Figures 2, 3, and 4, in total, 84 methyl esterified products were identified by GC/MS analysis and their parent components can be classified into MCAs, DCAs, BCAs, hydrocarbons (HCs), and other species (OSs). These products can also be divided into organochlorine compounds (OCCs, 40 such compounds) and nonorganochlorine compounds (NOCCs, 44 such compounds). Most of organochlorine compounds were from side reactions of coal oxidation with NaOCl aqueous solution.

As listed in Table S1, 28 MCAs were identified, including 12 alkanoic acids and 16 chloro-substituted alkanoic acids. For convenience, the methyl esterified products from SLL, SFB, and JCA oxidation with NaOCl are denoted as P_SLL_, P_SFB_, and P_JCA_, respectively. Most of these MCAs were detected in P_SLL_ and P_SFB_, whereas only 4 chloro-substituted alkanoic acids were detected in P_JCA_ (i.e., chloroacetic acid, dichloroacetic acid, 2,2-dichloropropanoic acid, and trichloroacetic acid).  Table S2 lists 11 DCAs detected in the products from coals oxidation, including 7 alkane-*α*,*ω*-dicarboxylic acids and 4 chlorine-substituted alkane-*α*,*ω*-dicarboxylic acids. Similar to the case of MCAs, most of these DCAs were only detected in P_SLL_ and P_SFB_.

Most detected species by GC/MS were BCAs. As listed in Table S3, 30 BCAs were identified in the products and 18 of them were chlorine-substituted BCAs. According to the number of carboxylic groups, the BCAs can be classified into 6 benzoic acids, 8 phthalic acids, 7 benzenetricarboxylic acids, 6 benzenetetracarboxylic acids, 3 benzenepentacarboxylic acids, and 1 benzenehexacarboxylic acid. All the BCAs were detected in P_SFB_, but only 16 and 14 were measured in P_SLL_ and P_JCA_, respectively.

Tables S4 and S5 list the 12 HCs and 3 OSs detected in the products, respectively. The 3 OSs consist of 1 tricarboxylic acid and 2 chlorine-substituted ethyl acetates. In addition, some unknown species were also detected (peak** X** in Figure S1) in P_SLL_, but their chemical structures were difficult to be identified. Heteroatoms such as S and O may account for the complexity of P_SLL_.

The yields of the above species were illustrated in Figure 2. The yields of MCAs decrease in the order: P_SFB_ ≫ P_JCA_ > P_SLL_, and most of MCAs detected in products are OCCs. In P_SFB_, trichloroacetic acid (peak** 9** in Figure S2) is the most abundant compound, accounting for ca. 50% of MCAs, while ca. 25% of MCAs are dichloroacetic acid (peak** 5** in Figure S2). The yields of DCAs decrease in the order: P_SLL_ ≫ P_SFB_ ≫ P_JCA_. In P_SLL_ the most abundant dicarboxylic acid is succinic acid (peak** 14** in Figure S1), accounting for ca. 40% of DCAs. Since *α*,*ω*-diarylalkanes are converted to DCAs via oxidation, the relatively higher yield of DCAs in P_SLL_ suggests that SLL is rich in *α*,*ω*-diarylalkanes structure. The yield of BCAs in P_SFB_ is close to that in P_JCA_, but significantly higher than that in P_SLL_. BCAs are yielded from aromatic clusters in coals via oxidation, thus a higher yield of BCAs in P_SFB_ and P_JCA_ implies that SFB and JCA contain more aromatic clusters than SLL. Considerable chlorine-substituted ethyl acetates were also yielded via SLL oxidation with NaOCl. From our previous report [23], these short chain chloro-substituted alkanoic acids (i.e., dichloroacetic acid and trichloroacetic acid) and chlorine-substituted ethyl acetates are derived from phenolic moiety of coals.

BCAs in various coals can be divided into 6 groups according to the number of carboxylic groups contained in BCAs mentioned above. The distribution of BCAs from coal oxidation is shown in Figure 3. As this figure shows, benzenetetracarboxylic acids are the most abundant compounds in BCAs for all coals. When the number of carboxylic groups contained in BCAs is less than 4, the yields of BCAs in P_SLL_ and P_SFB_ are higher than that in P_JCA_. On the contrary, when the number of carboxylic groups contained in BCAs is not less than 4, the yield of BCAs in P_JCA_ is higher than that in P_SLL_ and P_SFB_. The yield of benzenehexacarboxylic acid in P_JCA_ is several times higher than that in P_SLL_ and P_SFB_. The above experimental results imply that the yield of BCAs with more carboxylic group increases with coal rank. The BCAs are confirmed to be particularly useful for synthesizing functional material [14, 24] and medicines [25]. Murata et al. [26] reported that as the degree of condensation of the aromatic clusters increases, the number of carboxylic groups contained in the yielded BCAs increases. Thereby, our experimental results prove that high rank coals contain greater quantities of polycyclic aromatic compounds, while the lower condensed aromatic clusters, such as naphthalene and anthracene, are rich in low rank coals.

**Figure 5:**
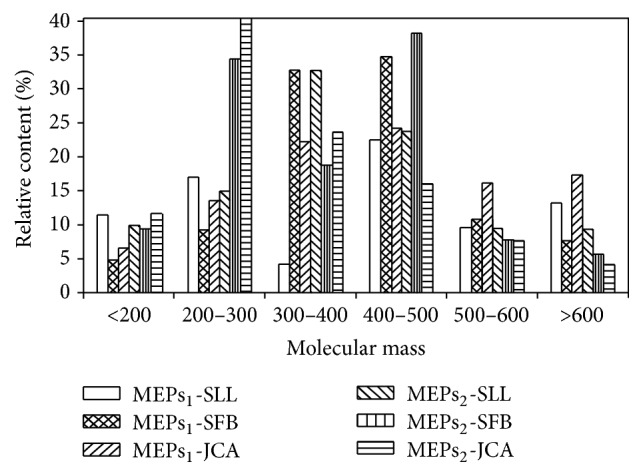
Distributions on molecular mass of the compounds identified with DART-TOF-MS.

The molecular mass distribution of detectable species in the esterified coal oxidation products acquired by GC/MS is between 60 and 340 Da, which only accounts for parts of components in the products. DART-TOF-MS expands the detection range according to molecular polarity and speeds up the analysis time with little or no sample pretreatment. As shown in Figure 4, most compounds distribute between 200 and 500 Da. There are 417, 348, and 327 compounds identified in MEPs_1_ of SLL, SFB, and JCA, respectively, and 369, 301, and 242 for MEPs_2_ of SLL, SFB, and JCA, respectively. Much more components were detected by DART-TOF-MS compared to GC/MS and the detection range of *m*/*z* was extended to around 800 Da. At the meantime, compounds from homologous series varying by 14 Da were also exhibited in each plot in Figure 4. Compared with the other two coal samples, less compounds were identified from the anthracite, which is consistent with the results from GC/MS. In previous report [10], associated ions including dimers and trimers were generated during the characterization of dried coal extracts using DART-MS. However, there is almost no associated ion identified by DART-MS from liquid coal derivatives. The detailed distributions of identified compounds according to molecular mass are shown in Figure 5. The analytical results from DART-MS are different from GC/MS because of various ionization mechanisms, and they can complement each other to explore molecular information of coal derivatives.

## 4. Conclusions

NaOCl aqueous solution is effective for coals oxidation. In this study, most organic compounds in coals were converted into soluble species through oxidation under mild conditions, even the Jincheng anthracite. Benzene polycarboxylic acids and chloro-substituted alkanoic acids were the major products from the reactions. Although most products from high rank coals were BPCAs, the products from lower rank coals consisted of considerable CSAAs. The yield of alkane-*α*,*ω*-dicarboxylic acids in Shengli lignite oxidation products was much higher, implying that Shengli lignite is rich in *α*,*ω*-diarylalkanes structure. In addition, as coal rank increases, the yield of BPCAs with more carboxylic increases, corroborating that high rank coals contain greater quantities of polycyclic aromatic compounds, while the lower condensed aromatic clusters, such as naphthalene and anthracene, are rich in low rank coals. GC/MS and DART-MS can complement each other to explore molecular information of coal derivatives.

## Figures and Tables

**Table 1:** Proximate and ultimate analyses (*W%*) of coal samples.

Coal samples	Proximate analysis			Ultimate analysis (daf)				
	*M*_ad_	*A*_d_	*V*_daf_	C	H	N	S	O_diff_
SLL	13.74	7.51	46.40	70.84	5.05	0.88	1.32	21.91
SFB	5.33	6.32	30.74	79.82	4.73	1.05	0.50	13.90
JCA	2.48	26.74	8.15	94.01	2.94	1.03	0.53	1.05

daf: dry and ash-free base; *M*_ad_: moisture (air dried base); *A*_d_: ash (dry base, i.e., moisture-free base); *V*_daf_: volatile matter (dry and ash-free base); diff: by difference.

**Table 2:** Yields of FC_1_ and IEF.

Coal samples	Yield (*W*%, daf)	
	FC_1_	IEF
SLL	1.4	trace
SFB	2.0	12.0
JCA	31.7	trace

## Nomenclature

AAs: Aliphatic acids
BPCAs: Benzene polycarboxylic acids
CSAAs: Chloro-substituted alkanoic acids
SLL: Shengli lignite
SFB: Shenfu subbituminous coal
JCA: Jincheng anthracite
GC: Gas chromatography
MS: Mass spectrometry
DART: Direct analysis in real time
TOF: Time-of-flight
FC_1_: Filter cake from the reaction mixture of SFB with NaOCl aqueous solution
F_1_: Filtrate from the reaction mixture of SFB with NaOCl aqueous solution
FC_2_: Filter cake from acidified F_1_
F_2_: Filtrate from acidified F_1_
ES_1_: Extraction solution from FC_2_ with CH_2_Cl_2_
ES_2_: Extraction solution from F_2_ with CH_2_Cl_2_
IEF: CH_2_Cl_2_-inextractable fraction from FC_2_
F_3_: Filtrate from dried ES_1_ and ES_2_ over anhydrous MgSO_4_
MEPs_1_: Methyl esterified products from distilled F_3_
IES: CH_2_Cl_2_-inextractable solution from F_2_
MEPs_2_: Methyl esterified products from distilled IES
MCAs: Monocarboxylic acids
DCAs: Dicarboxylic acids
BCAs: Benzene carboxylic acids
P_SLL_: Methyl esterified products of SLL oxidation with NaOCl
P_SFB_: Methyl esterified products of SFB oxidation with NaOCl
P_JCA_: Methyl esterified products of JCA oxidation with NaOCl
HCs: Hydrocarbons
OSs: Other species
OCCs: Organochlorine compounds
NOCCs: Nonorganochlorine compounds.

## Supplementary Materials

Supplementary Materials Three total ion chromatograms figures (Figures S1–S3) and five tables of products list (Tables S1–S5) detected by GC/MS are listed in the Supplementary Materials. Figure S1: total ion chromatograms of P_SLL_ (X stands for unknown species). Figure S2: total ion chromatograms of P_SFB_. Figure S3: total ion chromatograms of P_JCA_. Table S1: MCAs detected in the products from coal oxidation. Table S2: DCAs detected in the products from coal oxidation. Table S3: BCAs detected in the products from coal oxidation. Table S4: HCs detected in the products from coal oxidation. Table S5: OSs detected in the products from coal oxidation.
