# Supplementary material for: Molecular Distributions of Soluble Oxidation Products from Coals Characterized by Mass Spectrometers
Source: Int J Anal Chem. 2018 Apr 11;2018:5174172. doi: 10.1155/2018/5174172 (PMC5925153; doi:10.1155/2018/5174172)
Supplement: Supplementary Materials — Three total ion chromatograms figures (Figures S1–S3) and five tables of products list (Tables S1–S5) detected by GC/MS are listed in the Supplementary Materials. Figure S1: total ion chromatograms of PSLL (X stands for unknown species). Figure S2: total ion chromatograms of PSFB. Figure S3: total ion chromatograms of PJCA. Table S1: MCAs detected in the products from coal oxidation. Table S2: DCAs detected in the products from coal oxidation. Table S3: BCAs detected in the products from coal oxidation. Table S4: HCs detected in the products from coal oxidation. Table S5: OSs detected in the products from coal oxidation. [file 5174172.f1.pdf]

Supplemental Information

Table S1 MCAs detected in the products from coal oxidation

peak	MCAs	SLL	SFB	JCA
**1**	butanoic acid	×	×	
**2**	chloroacetic acid	×	×	×
**3**	2-chloropropanoic acid		×	
**4**	pentanoic acid	×	×	
**5**	dichloroacetic acid	×	×	×
**6**	2,2-dichloropropanoic acid	×	×	×
**7**	2-chlorobutanoic acid	×	×	
**9**	trichloroacetic acid	×	×	×
**10**	hexanoic acid	×	×	
**11**	2,2-dichlorobutanoic acid	×	×	
**13**	heptanoic acid	×	×	
**15**	2,3,3-trichloroacrylic acid	×	×	
**16**	2,2-dichloro-3-methylbutanoic acid		×	
**17**	2,2-dichloropentanoic acid	×	×	
**20**	3,5-dimethylheptanoic acid	×	×	
**22**	2,2,3-trichlorobutanoic acid	×	×	
**25**	2-chloroheptanoic acid		×	
**26**	2,2,3,3-tetrachloropropanoic acid	×	×	
**28**	nonanoic acid		×	
**29**	2,2,3-trichloro-3-methylbutanoic acid		×	
**32**	4-methylnonanoic acid	×	×	
**33**	decanoic acid	×	×	
**34**	2,2,3-trichloropentanoic acid		×	
**35**	2,2-dichlorooctanoic acid	×	×	
**41**	9-methyldecanoic acid	×		
**43**	dodecanoic acid	×	×	
**49**	tetradecanoic acid	×		
**59**	palmitic acid	×	×	

Table S2 DCAs detected in the products from coal oxidation

peak	DCAs	SLL	SFB	JCA
**14**	succinic acid	×		
**18**	2-methylsuccinic acid	×	×	
**19**	2-chloro-2-methylmalonic acid	×	×	
**23**	2-chlorosuccinic acid	×	×	
**24**	glutaric acid	×	×	
**27**	2,3-dichlorosuccinic acid	×	×	
**36**	heptanedioic acid	×	×	
**40**	2-chloro-3-(dichloromethyl)fumaric acid	×	×	
**45**	nonanedioic acid		×	
**47**	decanedioic acid		×	
**51**	undecanedioic acid	×	×	

Table S3 BCAs detected in the products from coal oxidation

peak	BCAs	SLL	SFB	JCA
**21**	benzoic acid	×	×	
**30**	3-chlorobenzoic acid		×	
**31**	4-chlorobenzoic acid		×	
**37**	2-chloro-4-methylbenzoic acid		×	
**39**	phthalic acid	×	×	×
**42**	isophthalic acid		×	
**44**	2,5-dichloro-4-methylbenzoic acid		×	
**46**	3-chlorophthalic acid		×	×
**48**	4-chlorophthalic acid		×	
**50**	4-chloro-5-methylphthalic acid	×	×	
**52**	4-chloro-5-ethylphthalic acid	×	×	
**53**	4-chloro-5-isopropylphthalic acid		×	
**54**	2,3,4-trichloro-5,6-dimethylbenzoic acid		×	
**55**	benzene-1,2,3-tricarboxylic acid	×	×	×
**56**	benzene-1,2,4-tricarboxylic acid	×	×	×
**57**	3,5-dichloro-4-methylphthalic acid		×	
**58**	benzene-1,3,5-tricarboxylic acid		×	
**62**	4-chlorobenzene-1,2,3-tricarboxylic acid	×	×	×
**63**	5-methylbenzene-1,2,4-tricarboxylic acid		×	
**64**	6-chlorobenzene-1,2,4-tricarboxylic acid	×	×	×
**65**	6-chloro-5-methylbenzene-1,2,4-tricarboxylic acid	×	×	
**66**	benzene-1,2,3,4-tetracarboxylic acid	×	×	×
**67**	benzene-1,2,3,5-tetracarboxylic acid	×	×	×
**68**	benzene-1,2,4,5-tetracarboxylic acid	×	×	×
**69**	5-chlorobenzene-1,2,3,4-tetracarboxylic acid	×	×	×
**70**	3-chlorobenzene-1,2,4,5-tetracarboxylic acid		×	×
**71**	5-chloro-6-methylbenzene-1,2,3,4-tetracarboxylic acid	×	×	
**74**	benzene-1,2,3,4,5-pentacarboxylic acid	×	×	×
**75**	6-chlorobenzene-1,2,3,4,5-pentacarboxylic acid		×	×
**77**	benzene-1,2,3,4,5,6-hexacarboxylic acid	×	×	×

Table S4 HCs detected in the products from coal oxidation

peak	HCs	SLL	SFB	JCA
**60**	icosane	×	×	
**61**	henicosane		×	
**72**	tricosane	×	×	×
**73**	tetracosane	×	×	×
**76**	pentacosane	×	×	×
**78**	hexacosane	×	×	×
**79**	heptacosane	×	×	×
**80**	octacosane	×	×	×
**81**	nonacosane	×	×	×
**82**	triacontane		×	
**83**	hentriacontane		×	
**84**	dotriacontane		×	

Table S5 OSs detected in the products from coal oxidation

peak	OSs	SLL	SFB	JCA
**8**	ethyl 2,2-dichloroacetate	×	×	×
**12**	ethyl 2,2,2-trichloroacetate	×	×	×
**38**	propane-1,2,3-tricarboxylic acid	×	×	

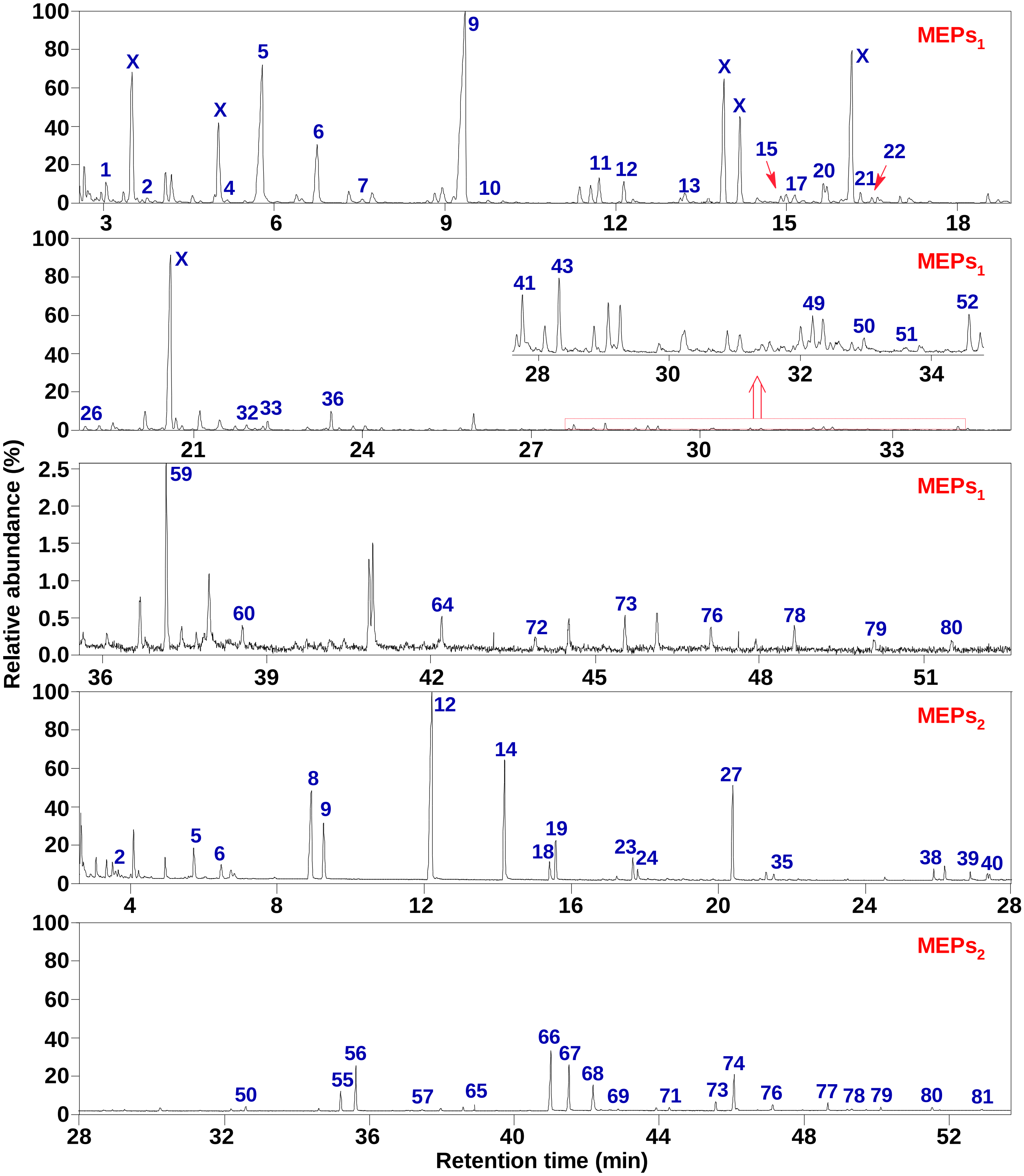

Figure S1 Total ion chromatograms of P_{SLL} (X stands for unkown species)

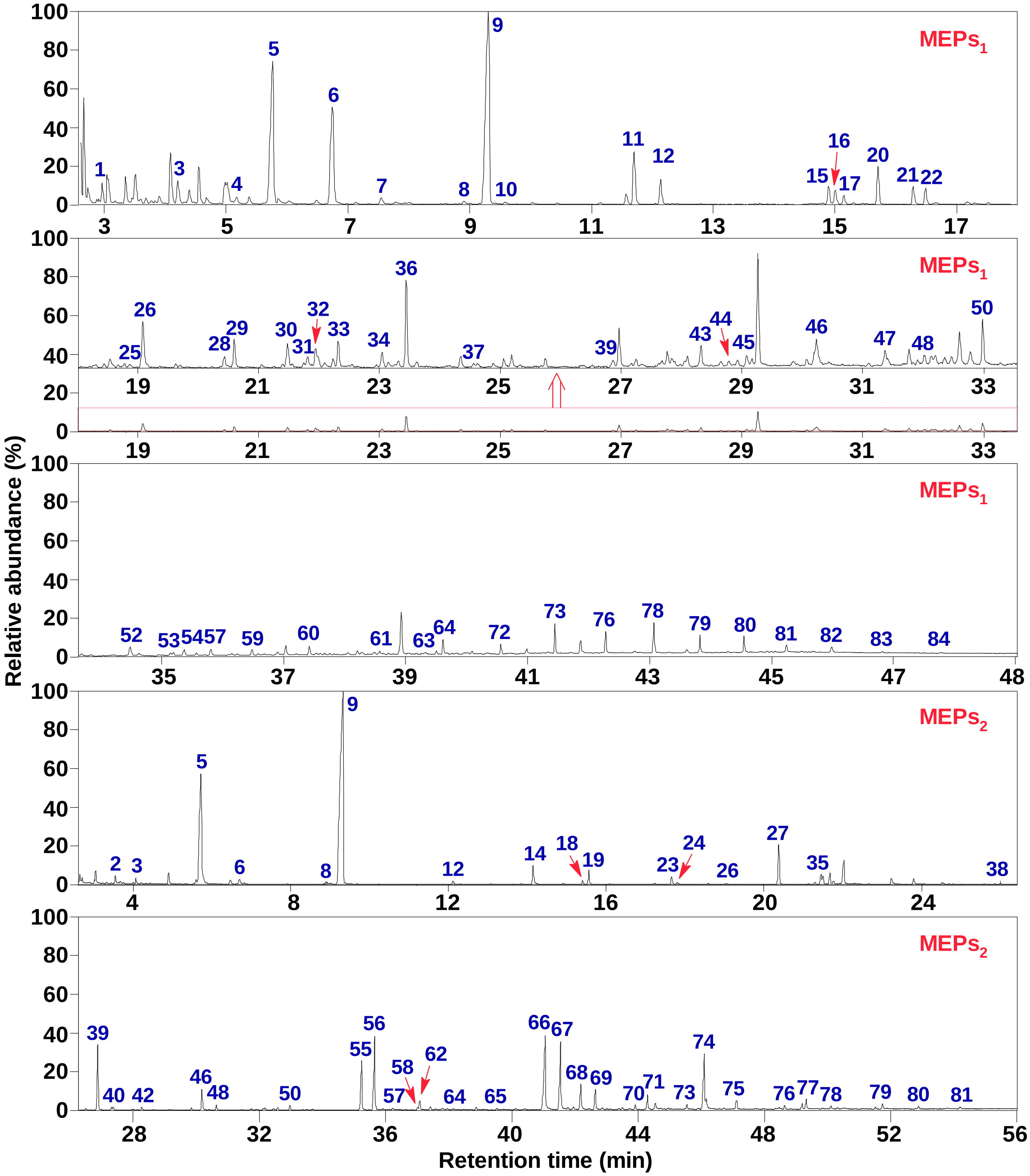

Figure S2 Total ion chromatograms of P_{SFB}

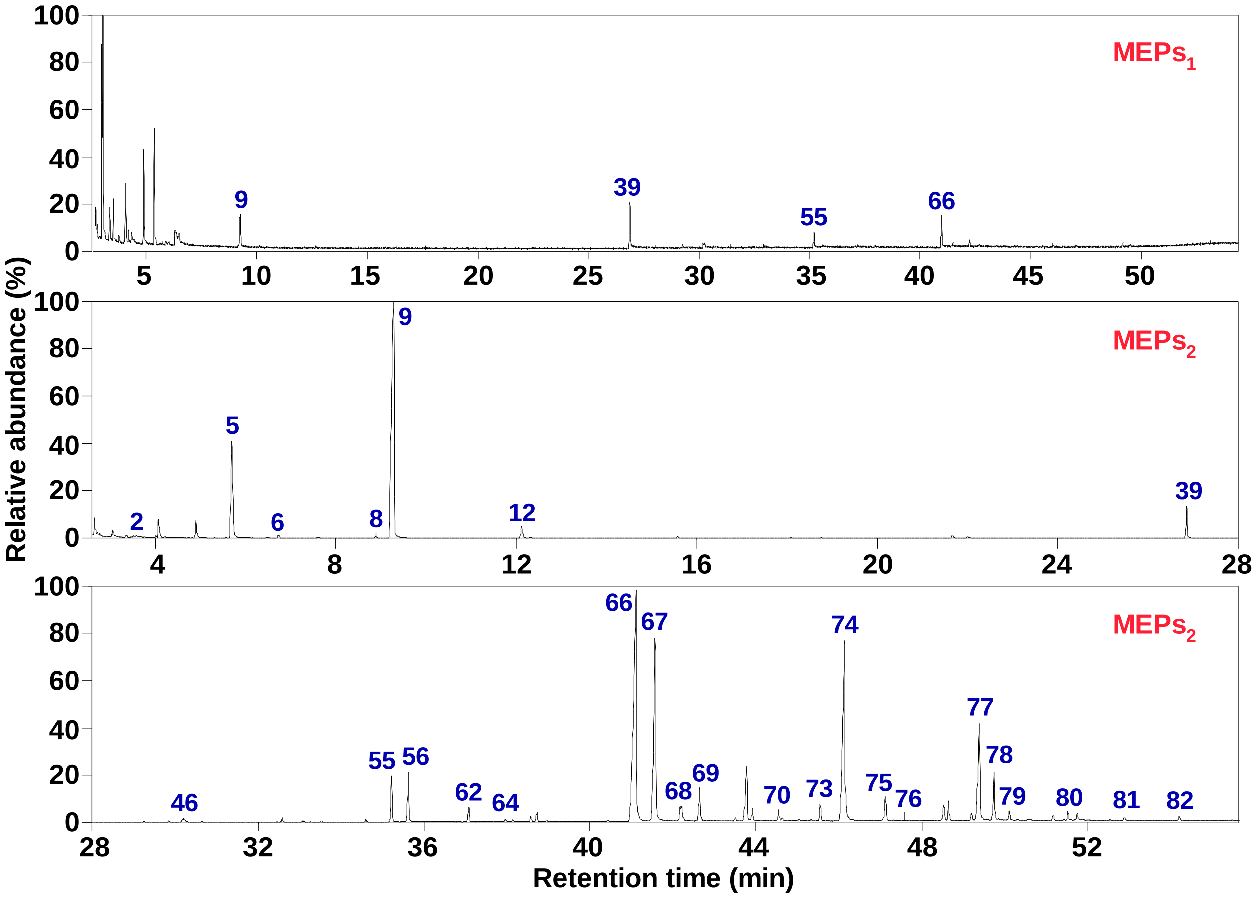

Figure S3 Total ion chromatograms of P_{JCA}
